# Supplementary material for: Modulating Weak Protein–Protein Cross-Interactions by the Addition of Free Amino Acids at Millimolar Concentrations
Source: J Phys Chem B. 2024 Jul 12;128(29):7199–207. doi: 10.1021/acs.jpcb.4c01086 (PMC11284779; doi:10.1021/acs.jpcb.4c01086)
Supplement: Supplementary file 1 — jp4c01086_si_001.pdf [file jp4c01086_si_001.pdf]

Supporting Information for Publication

Modulating weak protein-protein cross-interactions by addition of free amino acids at millimolar concentrations

Pamina M. Winkler[1], Cécilia Siri[1], Johann Buczkowski[2], Juliana V. C. Silva[2], Lionel Bovetto[2], Christophe Schmitt[2], Francesco Stellacci[1]*

[1]Laboratory of Supramolecular Nanomaterials and Interfaces, Ecole Polytechnique Fédérale de Lausanne (EPFL), Station 12, 1015 Lausanne, Switzerland.

[2]Nestlé Research, Nestlé Institute of Food Sciences, Vers-chez-les-Blanc, CH-1000 Lausanne 26, Switzerland

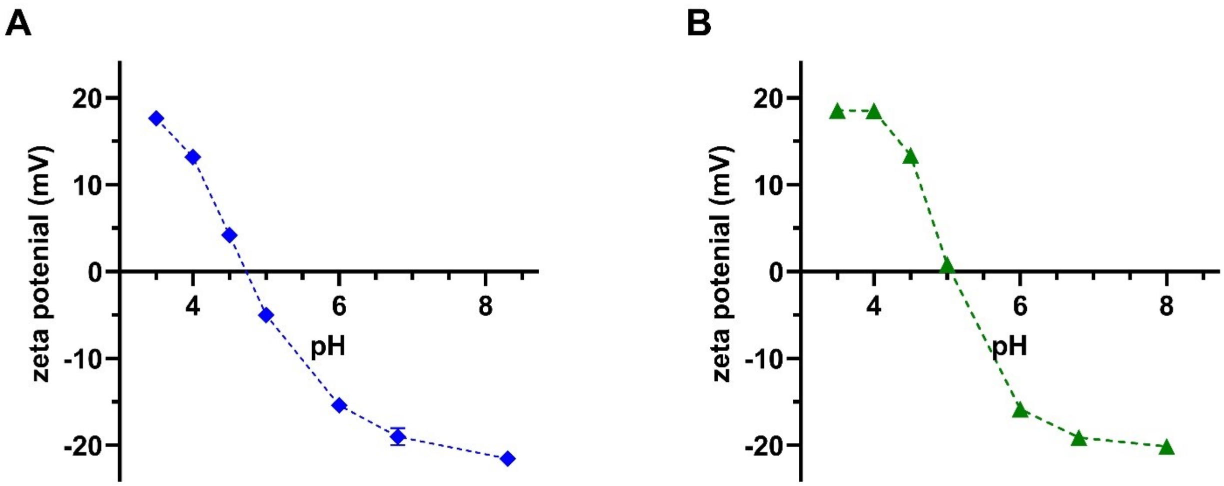

SI Figure 1: Measured zeta potential (averaged over 3 replica) for the two whey protein isolates WPI BLG (blue) and WPI ALAC (green) at a concentration of 5 mg/ml.

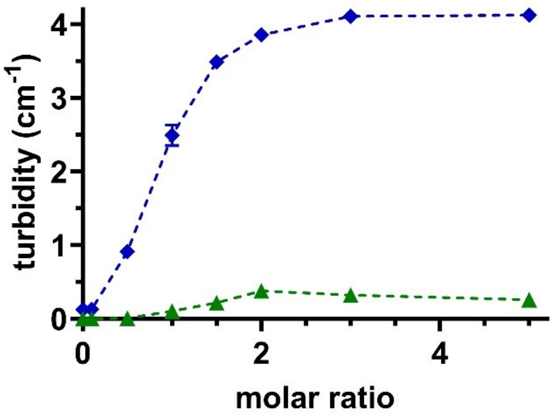

SI Figure 2: Measured turbidity measurements for the two whey protein isolates WPI ALAC (green) and WPI BLG (blue) interacting with lysozyme in 50mM sodium phosphate buffer (pH=6.9). The plotted values are averaged over two replica for each WPI. All stock solutions were filtered with a pore size of 0.2 µm. The final protein solutions were mixed to keep a constant final concentration of 0.25mM and to reach a molar ratio between [0.1-5]. To obtain the turbidity $\tau = 2.303 \cdot \frac{OD}{l}$ we measured the optical density OD at 600 nm using a spectrophotometer 30min after mixing the solutions. Note that the cuvette has a length $l = 1\ cm$.

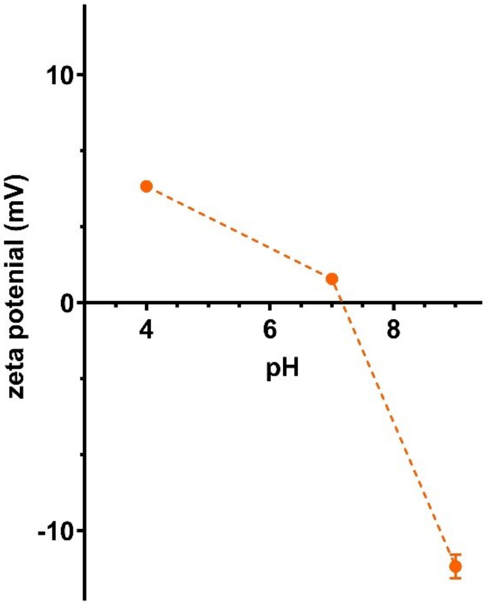

SI Figure 3: Measured zeta potential (averaged over 3 replica) for the canola protein isolate CPI NAP.

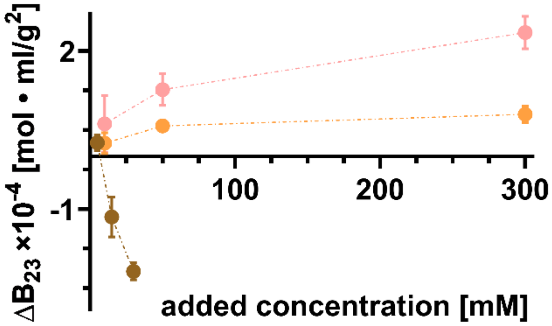

SI Figure 4: The change of B_{23} (ΔB_{23}) for the interaction between lysozyme grafted to the column and the whey protein isolate WPI BLG is displayed in presence of PEG 6k (brown), urea (pink) and betaine (orange) at different concentrations dissolved in the protein injected and in the 50 mM sodium phosphate buffer at pH ~6.9. The error bars reflect the measurement uncertainties (std of ΔB_{23}).

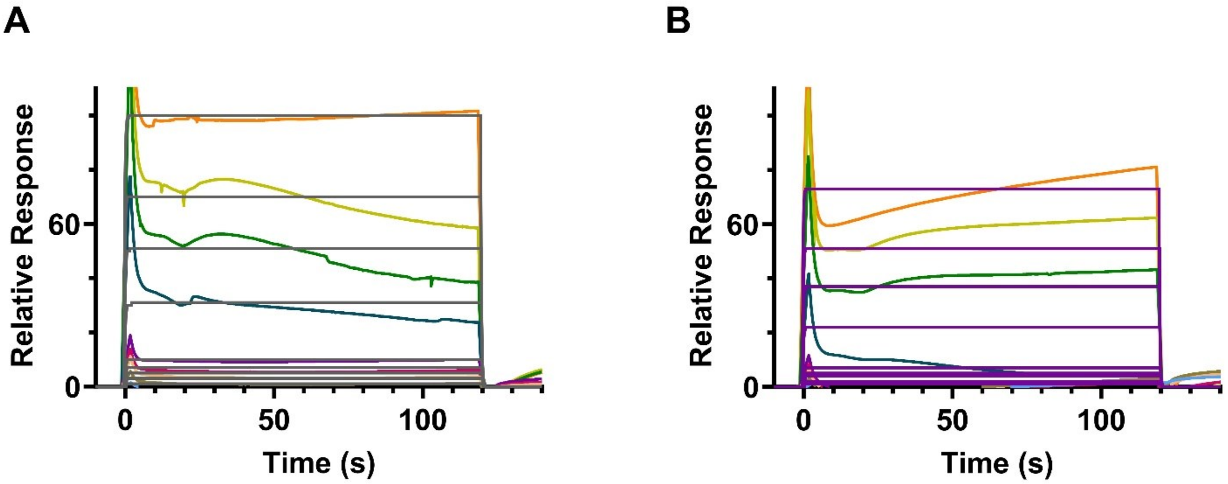

SI Figure 5: Representative SPR sensorgrams are shown for WPI BLG injections in the concentration range of 0.1-10 mg/ml in PBS **(A)** and in the presence of 10 mM arginine **(B)** interacting with lysozyme immobilized on the CM5 chip. The bulk response from the reference was subtracted.

Table S1. Averaged K_D values for the Lys-WPI BLG interaction in the presence of added amino acids. The reported K_D values obtained for the Lys-WPI BLG interaction in buffer and in the presence of added amino acids represent the average from >30 individual measurements on 5 different measurement days and 2 different CM5 sensor chips per condition

Lys-WPI BLG	in PBS	+ 10 mM proline	+ 10 mM glycine	+ 10 mM glycine + 10 mM arginine
Av. K_D ± Std [M]	$(2\pm1)\cdot10^{-03}$	(6±8)	(17±23)	(3±6)

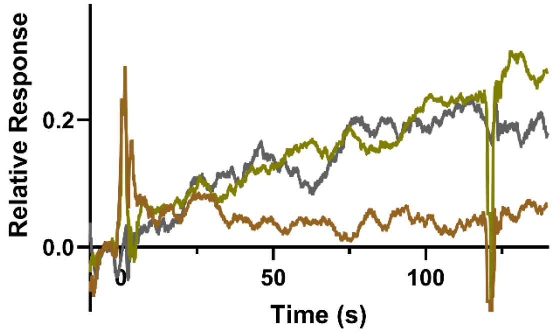

SI Figure 6: SPR sensorgram showing the response of PBS buffer (grey), and of proline (green) and glutamine (brown) added at a concentration of 100mM to the running PBS buffer flushed on immobilized Lys on the CM5 chip serving as “negative control”.
